# Psychosocial resources developed and trialled for Indigenous people with autism spectrum disorder and their caregivers: a systematic review and catalogue

**DOI:** 10.1186/s12939-020-01247-8

**Published:** 2020-08-06

**Authors:** Ian M. Shochet, Jayne A. Orr, Rachel L. Kelly, Astrid M. Wurfl, Beth R. Saggers, Suzanne B. Carrington

**Affiliations:** 1 School of Psychology and Counselling, Queensland University of Technology, Victoria Park Road, Kelvin Grove, QLD 4059 Australia; 2 The Cooperative Research Centre for Living with Autism (Autism CRC), Long Pocket, Brisbane, Queensland Australia; 3 School of Cultural and Professional Learning, Queensland University of Technology, Victoria Park Road, Kelvin Grove, QLD 4059 Australia

**Keywords:** Systematic review, Interventions – psychosocial/behavioural, Autism spectrum disorder, Neurodevelopmental disorder, Indigenous, First nations, Psychosocial wellbeing programs, Psychosocial wellbeing resources, Mental health

## Abstract

**Background:**

People with autism spectrum disorder (ASD) face many psychosocial challenges throughout life, highlighting the need for programs and resources promoting psychosocial wellbeing. Indigenous peoples with ASD and/or other neurodevelopmental disorders must overcome cultural and social barriers to access such supports. This study aimed to identify psychosocial programs and resources developed world-wide for this population by systematically reviewing research evaluating programs aiming to promote the psychosocial wellbeing of this population and/or their caregivers; and collating and reviewing resources developed to promote their psychosocial wellbeing.

**Methods:**

Searches were last conducted in December 2019. The systematic review searched 28 electronic databases, and 25 electronic databases were searched for resources promoting psychosocial wellbeing. Additional published and unpublished studies were identified from relevant reviews, authors of eligible articles, and experts working in Indigenous Health. Articles and resources were screened for inclusion using pre-defined criteria. Articles included in the systematic review were assessed for quality using the Mixed Methods Assessment Tool. The diversity and paucity of outcomes reported precluded pooling of study findings for meta-analysis.

**Results:**

Seven articles situated in the USA (2), Canada (3) and Australia (2); and eleven resources developed in Australia (9), Canada (1) and New Zealand (1) met inclusion criteria. All articles showed some promising findings for improving psychosocial wellbeing for Indigenous children with ASD and/or another neurodevelopmental disorder, and 5 of 7 evaluated the cultural adaptation of an existing evidence-based program for an Indigenous population. However, methodological quality was moderate or low (57% and 43% of articles respectively) and no studies had adult participants. The psychosocial wellbeing supports provided by the 11 resources included psychoeducation, community support, and services/workshops.

**Conclusions:**

Despite the paucity of research and resources found, important exemplars demonstrate that existing programs can be adapted to support Indigenous people with ASD and other neurodevelopmental disorders. While future policy should endeavour to facilitate Indigenous people’s access to support services, and encourage researchers to develop and evaluate programs promoting psychosocial wellbeing for this population, given complexities of designing and evaluating new programs, careful and appropriate cultural adaptations of existing evidence-based programs would increase feasibility of ongoing research without compromising outcomes.

## Background

The United Nations defines Indigenous peoples as the descendants of those who inhabited a country or a geographical region at the time when people of different cultures or ethnic origins arrived [1]. Any population that was/are the First People of their Nation will respectfully be referred to herein as Indigenous and in no way asserts that they are one group of people.

### Factors that impact the prevalence estimate of autism spectrum disorder (ASD) in Indigenous populations

The prevalence of ASD is increasing due to the expansion of the diagnostic criteria in 1994, better reporting practices, increased awareness of the disorder, increased diagnosis at an earlier age, and better access to services [2–5]. Although the prevalence of ASD worldwide has increased, prevalence in Indigenous populations is largely unknown, with rates often assumed to be equal to the national average [6, 7]. This lack of knowledge may create a problem of health inequity for Indigenous individuals and their caregivers. Hence, additional investigation is required to identify the prevalence and experience of Indigenous people with ASD, and to ascertain and address the gap in required services. Disability is a concept that people conceptualise in different ways depending on their cultural values, beliefs, and socio-historical context [8]. Research on the experience of disability in Aboriginal and Torres Strait Islander people in Australia shows that they do not have a word for disability, as it is inconsistent with their overarching values of diversity and inclusion [9]. As the Diagnostic and Statistical Manual of Mental Disorders (5th ed.; DSM 5) [10] is based on a Western, medical, deficit model, it has been argued that this model may not be applicable to, or accepted by, Indigenous peoples due to the discordance with Indigenous cultural values of diversity and inclusion [9]. This impacts prevalence estimates as Indigenous people who do not consider their diversity as a disability are less likely to seek out a diagnosis.

In Australia, culture and language barriers contribute to the under-diagnosis of ASD within the Indigenous population [11]. Some characteristics, such as avoiding eye contact, may not be considered problematic in Indigenous cultures [12]. Diagnostic and assessment tools currently being used lack cultural sensitivity [13, 14]. Stereotyping and ethnic biases are another obstacle to accurate diagnosis. Indigenous children in Australia are reported as more likely to receive a diagnosis of intellectual disability than any other developmental disorder [11, 15]. In some ethnic groups, it appears that professionals are less likely to consider comorbid developmental disorders if the child already has a diagnosis of intellectual disability [16].

Accurate understanding of prevalence is also limited by accessibility and cultural sensitivity of services that provide accurate diagnoses of ASD. Indigenous people living in remote areas have limited access to assessment and diagnostic facilities [15, 17]. In addition to limited access, the history of oppression and colonisation may discourage people in Indigenous communities from seeking help from government services [18]. When Indigenous people do engage with support services, they often report feelings of intimidation and that they had not felt welcomed or heard [19].

As previously established, prognosis improves with early diagnosis and treatment [20–22]. Current best practice recommends that behavioural and cognitive interventions are used in conjunction with psychotropic medication as the primary treatment for ASD characteristics [23, 24]. Due to the persistent nature of ASD characteristics, the difficulties experienced by this population rarely improve without support [25]. As there are a number of barriers that prevent Indigenous people from accessing an accurate diagnosis, it is probable that there are a number of Indigenous people with ASD characteristics who are denied appropriate and timely treatment.

### Psychosocial challenges across developmental stages

Accurate diagnosis and access to appropriate resources is important for providing support at each developmental stage. The overarching psychosocial challenges experienced by Indigenous people with ASD tend to be present across the lifespan (e.g., comorbid diagnoses and difficulties with social relationships). These challenges and their associated consequences do however manifest differently for children, adolescents and adults, and can be complicated by psychosocial difficulties encountered at different developmental stages. There is therefore a need for resources and support programs for people with ASD throughout the lifespan [26].

Children with ASD are more likely than their neurotypical peers to receive a comorbid diagnosis of a depressive disorder, anxiety disorder, obsessive-compulsive disorder, or Attention-Deficit/Hyperactivity Disorder (ADHD), which is associated with decreased wellbeing of parents and siblings, increased problems with social skills, and decreased quality of life [27]. Some children with ASD are less emotionally ready to start school [28]. Difficulties with social interactions, when combined with the expression of the core ASD characteristics (e.g., repetitive and restricted behaviours and thoughts, lack of assertiveness, heightened behavioural reactions), can increase the likelihood of peer rejection, with 63% of children with ASD falling victim to bullying [29]. The transition to adolescence is associated with increased pressure to develop social connections, but the psychosocial challenges of ASD can reduce opportunities to develop social skills, reduce capacity to build and maintain friendships, and perpetuate feelings of social isolation and loneliness [30–33]. Adolescents with ASD have a higher risk of developing comorbid depression [34, 35]. Affect regulation, the unconscious or conscious strategies that a person uses to change the intensity or duration of their emotional experience, presents another challenge for adolescents with ASD [36, 37]. Adolescents with ASD are more likely to use maladaptive techniques, such as avoidance and venting [38], defence and crying [39], averted eye contact [40], suppression [41], and blaming of others [42]. The effects of comorbid psychopathology during adolescence can persist into adulthood, and negatively impact on the wellbeing of adults with ASD [27, 43]. Adults with ASD and comorbid depression are less likely to engage in and complete tertiary education [44, 45], and characteristics of ASD and comorbid psychopathology can provide barriers to employment [46, 47]. The initiation and maintenance of romantic and sexual relationships can also be difficult for people with ASD due to deficits in social skills and concerns about fulfilling relationship expectations [48].

### Supporting caregivers of people with ASD

A multi-level ecological approach acknowledges that people with ASD are supported by a number of people at different proximities, including parents, family members, foster carers, tutors, teachers, schools and communities [49] referred to as caregivers from hereon. Difficulties experienced by children and young adolescents with ASD can lead to heightened stress and conflict for caregivers [50]. Research into the broader autism phenotype demonstrates a modest hereditary link in the diagnosis of ASD [51], with siblings, parents, and/or grandparents commonly exhibiting a milder form of ASD characteristics. Parents who display the broader autism phenotype report higher levels of stress and depression than norms [52]. A range of mental health and wellbeing programs have demonstrated positive psychosocial outcomes for parents and siblings of children and young people with ASD [53–55].

Working with students with ASD can also present unique challenges for schools and teachers. Students with ASD are significantly more likely than their neurotypical peers to require educational, vocational, or social support services [56]. However, many teachers report that they are underprepared and lack the confidence to provide the support that students with ASD require in the classroom [57].

### This study

Many of the existing interventions for people with ASD include behavioural approaches that focus on developing cognitive, communication, and language skills rather than addressing the aforementioned psychosocial challenges that many people with ASD experience [28, 51, 58]. The psychosocial challenges experienced across the lifespan by Indigenous people with ASD and their caregivers highlight the need for programs (i.e., supportive programs, workshops, interventions or strength-based therapy) and strength-based resources (e.g., psychoeducation, community support platforms, and services) that promote the psychosocial wellbeing of this population. We adopt the definition of psychosocial wellbeing that encompasses emotional wellbeing (feelings of positive affect such as happiness and hopefulness, life satisfaction, and quality of life), psychological wellbeing (positive functioning, self-acceptance, personal growth, resilience, and fulfilment), and social wellbeing (connectedness, feeling valued by society, thriving in social relationships, and/or enjoying spiritual health) [59].

This study was initially intended to focus on the population of Indigenous people of any age who display characteristics, or have a diagnosis, of ASD. However, our initial review of the literature found a dearth of information for Indigenous people with ASD. We therefore expanded our search to include all neurodevelopmental disorders in order to elicit research on programs and resources that might either be more generically applicable, able to be readily adapted for an ASD population, or serve as a model for future research in this area. Hence, this study will focus on the population of Indigenous people of any age who display characteristics, or have a diagnosis, of ASD and/or another neurodevelopmental disorder categorised in DSM 5 [10]. This study will aim to discover which psychosocial programs and resources have been developed anywhere in the world to support this population by:

1. systematically reviewing research articles evaluating programs developed and trialled with the aim of improving psychosocial wellbeing for this population and/or their caregivers, and
2. collating and reviewing resources (including programs, support services, community services, community groups, and online resources) developed to boost psychosocial wellbeing for this population and/or their caregivers.

## Methods

### Systematic review of psychosocial programs

The systematic review was conducted according to the procedures outlined in the Preferred Reporting Items for Systematic Review and Meta-analysis (PRISMA) statement [60].

#### Search strategy

The search strategy was designed to detect studies relevant to Indigenous people (e.g., Aboriginal, Maori, Native American, First Nations) with characteristics or a diagnosis of a neurodevelopmental disorder (e.g., ASD, intellectual disability, specific learning disorder, ADHD) that described a support (e.g., therapy, program, resource, intervention) designed to improve psychosocial wellbeing. No publication status restrictions were applied. Further, no language restrictions were applied as the researchers have had past success translating the text of non-English articles into English using *Google Translate* (Google, Mountain View, California), and contacting authors of translated articles for clarification when required. A standard search string was used for most databases; however, where search space was limited, one of two simplified search strings was used (see Tables 3 and 4 in Appendix 1). When unable to use the simplified search strings, one-word searches were conducted. The detailed searches are described in Tables 5 and 6 in Appendix 2. A research librarian was consulted when developing the strategy in order to ensure the appropriateness of its scope.

Electronic searches covered 25 databases of peer reviewed, published research; and three grey literature databases (Open Grey, Open Access Theses and Dissertations, and ProQuest Dissertations and Theses Open) (see Appendix 3). As the researchers’ interest and work in this area increased over time, searches were conducted initially on the 12th and 20th of December 2017, and were repeated three times, with the final searches conducted on the 26th and 27th of November 2019. A combination of subject headings and keywords were used in all searches, including Emtree headings and MeSH terms in EMBASE and PubMed respectively. In addition, reference lists of eligible articles and related reviews were examined to locate studies that potentially met the search criteria, and researchers in the field and members of organisations providing support to Indigenous people with characteristics or a diagnosis of ASD and/or other neurodevelopmental disorders were contacted by email to request details of additional studies that met the inclusion criteria.

#### Study eligibility criteria

Articles were deemed eligible if they described: (a) an intervention, support, initiative or strength-based therapy that had been developed and trialled (b) which aimed to improve the psychosocial, emotional, mental, and/or spiritual health, wellbeing, resilience, adaptive functioning, or connectedness of (c) Indigenous children, adolescents, and adults who (d) displayed characteristics of, or were diagnosed with, ASD or any other neurodevelopmental disorder listed in current or previous versions of the DSM 5 [10], such as Asperger syndrome, childhood disintegrative disorder, and pervasive developmental disorder not otherwise specified (PDD-NOS), (e) and/or their caregivers. Articles in any language, conducted anywhere in the world, with any design, and from any date were considered eligible. In order to focus on programs promoting psychosocial wellbeing, articles that described pharmacological or educational interventions, supports, or outcomes were excluded.

#### Study quality assessment

The methodological quality of studies extracted for the systematic review was assessed independently by two reviewers (JO and RK) using the Mixed Methods Appraisal Tool (MMAT), version 2018 [61]. The MMAT provides a checklist of criteria relating to study design and selection bias, sample size, data collection methods, intervention integrity, and data analysis and interpretation for appraising the quality of quantitative, qualitative and mixed methods studies included in systematic reviews. It has been found to be reliable, efficient and to have demonstrated content validity [62–64]. The reviewers’ findings were compared and discussed, with discrepancies resolved by consensus. For each study, the percentage of methodological quality criteria endorsed guided whether the study quality was assessed as “high” (≥ 80%), “moderate” (41–79%) or “low” (≤ 40%).

#### Data extraction and data synthesis

The results from all searches were exported into the EndNote (version X8) citation manager where duplicates were removed. Data extracted from eligible articles included authors, year published, study setting/location, publication type, description of the program, number of participants, study design, study findings or outcomes, and research strengths and limitations. The diversity and paucity of outcomes reported precluded the pooling of study findings for meta-analysis. Instead, findings were summarised using a narrative approach.

### Catalogue of psychosocial resources

#### Search strategy

The search strategy was designed to identify psychosocial resources developed to improve the psychosocial wellbeing of Indigenous people (e.g., Aboriginal, First Nations, Maori, Native American) with neurodevelopmental disorders (e.g., ASD, Asperger syndrome, intellectual disability). A research librarian was consulted when developing the strategy in order to ensure the appropriateness of its scope and efficacy. No language restrictions were applied. The search string used in all database searches was: (Indigenous OR “First Nations” OR Aborigin* OR “First Nation” or Aboriginal) AND (Asper* OR Autis* OR “Neurodevelopmental Disorder” OR “Autism Spectrum Disorder”). The delimiters (Incidence OR Prevalence OR Rates) were utilised to exclude such studies in order to increase the efficacy of search string output.

Electronic searches covered 21 databases of peer reviewed, published research; and four grey literature databases (Google Scholar, Microsoft Academic, Advanced Google search engine and Quick Find from the Queensland University of Technology library) (see Appendix 4). As the researchers’ interest and work in this area increased over time, searches were conducted initially on the 12th and 20th of December 2017, and were repeated three times, with the final searches conducted on the 3rd and 4th of December 2019. In addition, reference lists of eligible articles and related reviews were examined to locate studies that potentially met the search criteria; and authors of the studies included in the systematic review described in this manuscript, researchers working in the field of Indigenous Health Research, and members of organisations providing support to Indigenous people with a diagnosis or characteristics of ASD and/or other neurodevelopmental disorders were contacted by email to request details of additional studies (regardless of publication status) that met the inclusion criteria. The developers and co-ordinators of the resources included in the review were also contacted.

#### Study eligibility criteria

Resources were deemed eligible if they described: (a) a psychosocial, mental health and/or strength-based resource that was developed or trialled for implementation with (b) Indigenous children, adolescents or adults who (c) displayed characteristics of, or were diagnosed with, ASD or any other neurodevelopmental disorder listed in current or previous versions of the DSM 5 [10], such as Asperger syndrome, childhood disintegrative disorder, and pervasive developmental disorder not otherwise specified (PDD-NOS), (e) and/or their caregivers. Resources in any language, from anywhere in the world, with any design, and from any date were considered eligible.

#### Data extraction

The results from all searches were exported into the EndNote (version X8) citation manager where duplicates were removed. The titles and abstracts of the remaining articles were manually screened, and ineligible articles removed. For articles that remained, the full texts were assessed against the selection criteria.

## Results

### Systematic review

**Fig. 1:**
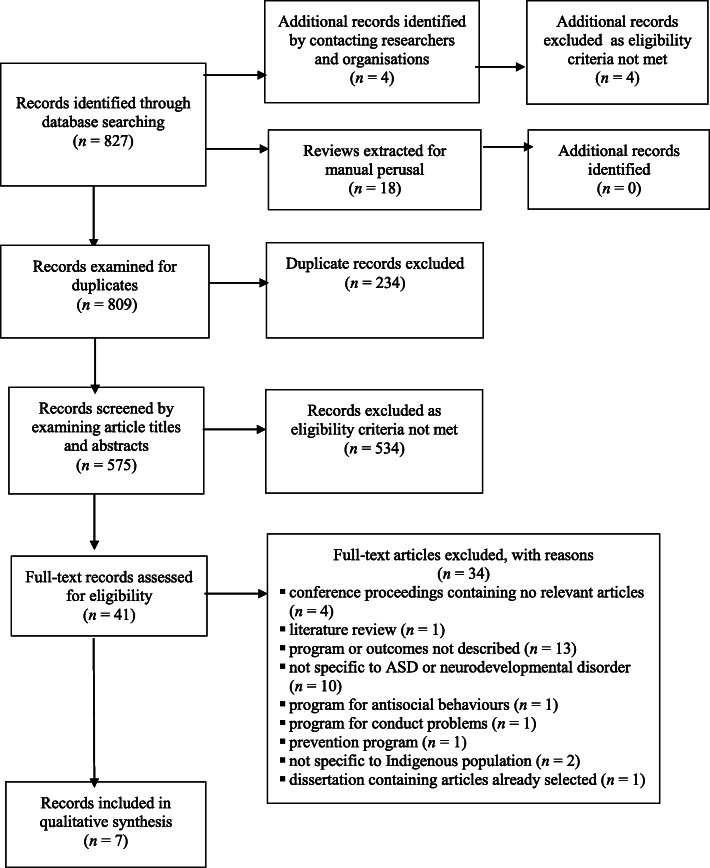
PRISMA flow diagram showing the article selection process for the systematic review

Figure 1 illustrates the article selection process for the systematic review. Database searches returned 827 records (hereafter referred to as articles), 18 of which were reviews, leaving 809 potential articles, all written in English. The reference lists of the 18 reviews were manually perused for articles that met the eligibility criteria, with no additional articles identified. Contacting researchers and organisations yielded 4 additional articles, all of which were excluded as they did not meet the eligibility criteria. Examining the 809 articles for duplicates resulted in 234 duplicates being removed. The remaining 575 articles were screened independently by 3 researchers, and discrepancies were discussed until consensus was reached. The first level of screening involved examining article titles, reading their abstracts when further information to assess eligibility was required, and resulted in the exclusion of 534 articles. The full texts of the remaining 41 articles were read to assess eligibility for inclusion, with 34 articles excluded. Hence, seven articles met the eligibility criteria.

The seven articles that met the eligibility criteria (see Table 1) were derived from 5 studies reported in 5 peer-reviewed journal articles and 2 dissertations. Over half of the articles used a qualitative design (*n* = 4, 57%), with the remaining articles employing a mixed-methods (*n* = 2, 29%) or a quantitative before and after design (*n* = 1, 14%). Quality assessment rated the quality of the majority of articles (*n* = 4, 57%) as moderate, and the quality of the remaining articles (*n* = 3, 43%) as low.

**Table 1:** Summary of studies that met the criteria of the systematic review

Author Date (Study location)	ID	Type and title	Description and aim	Study design, participants, and quality (MMAT)	Findings	Strengths and limitations
Bettag, 2016 (Hawaii) [65]	A	Dissertation: Analysis of the adaptation of the responsive teaching paradigm to serve predominantly native Hawaiian communities: A framework for guiding culturally appropriate, family-centered, relationship-based early childhood services.	Adapted and implemented a play-based, relationship-oriented program in low SES Native Hawaiian communities to strengthen caregiver-child relationships and improve children’s developmental functioning.	Mixed methods naturalistic before and after design. *N =* 44 Children (0–5 years) with developmental delay and their families. Quality: Moderate.	Significant increase in children’s post program developmental outcomes across personal, social, cognitive, and communication domains. Significant improvements were seen in the relationship between caregiver and child after completing the program. Caregivers reported a high degree of satisfaction with the program and its benefits.	Each family received an in-home program of 24 weekly sessions tailored for their strengths and needs. Used validated pre and post scales and semi-structured assessments. Non-randomised single group study. Emphasis on naturalistic validity and accessibility decreased experimental control and undercut causal relationship between program completion and outcomes. Errors in coding of pre and post interaction videos led to lower inter-rater reliability of semi-structured assessments. Findings were derived from the quantitative data only. Attrition bias (61% attrition) threatens study validity.
Keightley et al., 2018 (Canada) [66]	B	Article: Investigating a theatre-based intervention for Indigenous youth with Fetal Alcohol Spectrum Disorder (FASD)	Used theatre-based activities (e.g., voice work, breathing, group dynamics) to facilitate social and emotional awareness of Indigenous children with developmental impairment associated with FASD.	Qualitative exploratory case-series design. *N* = 3 Children (9–14 years) with FASD or alcohol-related neurodevelop-mental disorder. Quality: Moderate.	Qualitative data from participants, parents, and program facilitators 2 weeks post-program showed improvements in self-esteem, social skills, and emotional awareness.	Included culturally relevant activities in the program (e.g., medicine wheel crafting, drumming). Non-randomised single group study Only anecdotal data was gathered from focus group follow up interviews, and not all participants contributed to the data. Small sample size.
Lindblom, 2017 (Canada) [67]	C	Article: ‘It gives them a place to be proud’- Music and social inclusion. Two diverse cases of young First Nations people diagnosed with autism in British Columbia, Canada.	Used Indigenous music as a tool to promote a sense of inclusion in Indigenous children with ASD.	Qualitative ethnography and Indigenous research methods design. *N* = 2 Children with Indigenous status and ASD (1 boy: 8 years old; 1 girl: 16 years old). Quality: Low.	Qualitative data suggested that traditional and contemporary music can be used to facilitate inclusion for Indigenous children with ASD through increased connection with people around them, including increased eye contact, singing, and playing of instruments together.	Researcher collaborated with traditional Elders and knowledge holders. Gathered data from semi-structured interviews, observations, field notes, and videos. Conducted follow up interviews 12 months post intervention to strengthen interpretation of results. Non-randomised single group study. Small sample size.
Lindblom, 2017 (Canada) [68]	D	Article: Exploring autism and music interventions through a First Nations lens.	Qualitatively explored the meaning, purpose and use of music for First Nations children with ASD.	Qualitative ethnography and Indigenous research methods design. *N* = 5 Children with Indigenous status and ASD (4 boys: 6, 8, 12 and 15 years old; 1 girl: 16 years old). Quality: Low.	Qualitative data suggested that music can improve mood, communication, relaxation and focus during study for First Nations children with ASD.	Researcher collaborated with traditional Elders and knowledge holders. Gathered data from semi-structured interviews, observations, and videos. Conducted follow up interviews 12 months post intervention to strengthen interpretation of results. Non-randomised single group study. Small sample size.
Najera, 2012 (USA) [69]	E	Dissertation: Adaptive behavioural analysis (ABA) in Native American homes: A culturally responsive training for paraprofessionals.	Developed and evaluated a culturally adapted 3 module training resource for ABA tutors providing home-based support for Indigenous children with ASD.	Qualitative design. *N* = 3 Mental health professionals with experience of ABA and who worked with Native American populations. Quality: Moderate.	Evaluators concluded that the resource required further development but could be used to support ABA tutors working with Native American families.	Developed a culturally responsive training resource for ABA delivery in Native American homes. Evaluators had experience of ASD and working with Indigenous people. Non-randomised single group study. Small sample size. Only one evaluator identified as Indigenous.
Wagner et al., 2019 (Australia) [70]	F	Article: Improving self-regulation and executive functioning skills in primary school children in a remote Australian Aboriginal community: A pilot study of the Alert Program®.	Piloted and adapted an 8-session weekly teacher-delivered self-regulation program in a rural Australian Indigenous community with a high prevalence of developmental impairment associated with FASD to improve students’ emotion regulation and executive functioning skills.	Quantitative before and after design. *N* = 25 Children in years 1 to 5 who had attended at least 20% of school over the past 6 months with or without a diagnosis of FASD. Quality: Low.	Parents/caregivers and teachers reported a significant improvement in students’ emotion regulation and executive functioning. Clinical improvements were more commonly reported by parents than teachers (executive functioning 33.3% vs. 26.1%; emotion regulation: 54.5% vs. 17.4%).	Collaborated with Aboriginal Elders and community, school staff, teachers and Aboriginal and Islander Education Officers to adapt and implement the program. Teachers were trained to deliver the program and were supported during delivery. Used validated scales to gather data from teachers and parents at 3 time points (pre, post and 8-week follow-up). A measure of disruptive behaviour was used as a proxy measure for emotion regulation. Non-randomised single group study. Non-blinded intervention delivered to the whole class. Small sample size. Attrition bias (52% attrition) threatens study validity.
Wagner et al., 2019 (Australia) [71]	G	Article: RE-AIM evaluation of a teacher-delivered programme to improve the self-regulation of children attending Australian Aboriginal community primary schools.	Analysis of teachers’ experience of implementing an 8-session weekly teacher-delivered self-regulation program in a rural Australian Indigenous community with a high prevalence of developmental impairment associated with FASD to improve students’ emotion regulation and executive functioning.	Mixed methods design. *N* = 29 Classroom teachers from 8 primary schools in a rural Australian Indigenous community. Quality: Moderate.	Teachers reported increased understanding of self-regulation and the Alert Program®, greater self-efficacy in managing student needs and behaviours, and inclusion of several positive changes to their teaching and behaviour management practices.	Used a theoretically based evaluation framework to gather self-report data. All teachers attended one of two training sessions. 21% of teachers did not attend both training sessions. No independent fidelity checks: teacher self-reports of program implementation may have been biased by teachers’ motivation to portray full acceptance of the program. Qualitative feedback was not actively sought from teachers, limiting understanding of teachers’ experience. Attrition bias (41% attrition) threatens study validity.

All articles identified by the systematic review reported success across a range of psychosocial outcomes. The studies described in six of the articles showed positive outcomes associated with the implementation and evaluation of programs designed specifically for Indigenous people with ASD, including improvements in personal (e.g., mood, self-esteem, relaxation, self-regulation, executive functioning; see Table 1, Articles A, B, D, F, & G), social (e.g., social skills, emotional awareness, eye contact; Articles A - C), cognitive (e.g., concentration; Articles A & D), and communication outcomes (Articles A & D). The seventh article (Article E) reported favourable evaluations of a resource designed to assist with the cultural adaptation of an existing program when working with Indigenous children with ASD and their families.

Bettag (Article A) [65] described a program that aimed to improve the developmental (cognitive and communication) outcomes of young children with developmental delay by strengthening parent/caregiver-child relationships and enhancing parenting and teaching skills, and used validated self-report scales and semi-structured observational assessments to measure outcomes. Other articles, Keightley et al. (Article B) [66] and Lindblom (Articles C & D) [67, 68] used theatre-based activities or traditional and non-traditional music in programs with Indigenous children with developmental impairment or ASD. According to observational reports from researchers, and anecdotal evidence gathered from interviews with participants and caregivers, the programs achieved increased inclusion, improved mood, and increased relaxation in participants. Three of the seven articles described programs that had been adapted for use with Indigenous populations. Najera (Article E) [69] evaluated the effectiveness of a resource that aimed to support tutors to culturally adapt an Adaptive Behavioural Analysis (ABA) program when working with Indigenous children with ASD and their families. The evaluation of the resource by three practitioners experienced in using ABA and working with Indigenous children and families found that the training modules required further development but were considered to be effective for promoting tutors’ awareness of cultural competency, guiding their exploration of their own cultural beliefs, and informing them of the cultural beliefs and values of Native American homes, which could potentially make them more effective practitioners. Wagner et al. (Articles F & G) [70, 71] adapted the classroom-based Alert Program® (an evidence-based program that improves emotion regulation and executive functioning but had not been investigated in Indigenous communities) for a teacher-delivered curriculum in a rural Australian community that was affected by high rates of developmental impairment associated with Fetal Alcohol Spectrum Disorder (FASD). Pilot results (Article F) showed improvements in student outcomes as reported by parents and teachers. Evaluation of teachers’ experience of delivering the adapted Alert Program® in a larger trial across eight schools in a rural Australian community (Article G) revealed significant improvement in teachers’ understanding of children’s self-regulation, and positive improvements in their teaching and behavioural management of students who had developmental impairment associated with FASD and emotion regulation impairments.

All articles included in the systematic review were published within the last 10 years, described research conducted in North America (3 in Canada, 2 in the USA) and Australia (2); and focused on supporting children: 3 articles focused on ASD (Articles C - E), 3 articles focused on developmental impairment associated with FASD (Articles B, F & G), and 2 articles included children with developmental delay comorbid with other developmental disorders (Articles A & B). This review only identified five articles published in peer-reviewed journals (Articles B - D, F & G), with the remaining articles (A & E) contained in doctoral dissertations. Furthermore, the 7 articles in this systematic review reported findings from only 5 studies: (Articles C & D were derived from one dissertation, and Articles F & G reported findings from a multi-phase research project).

### Catalogue of psychosocial resources

**Fig. 2:**
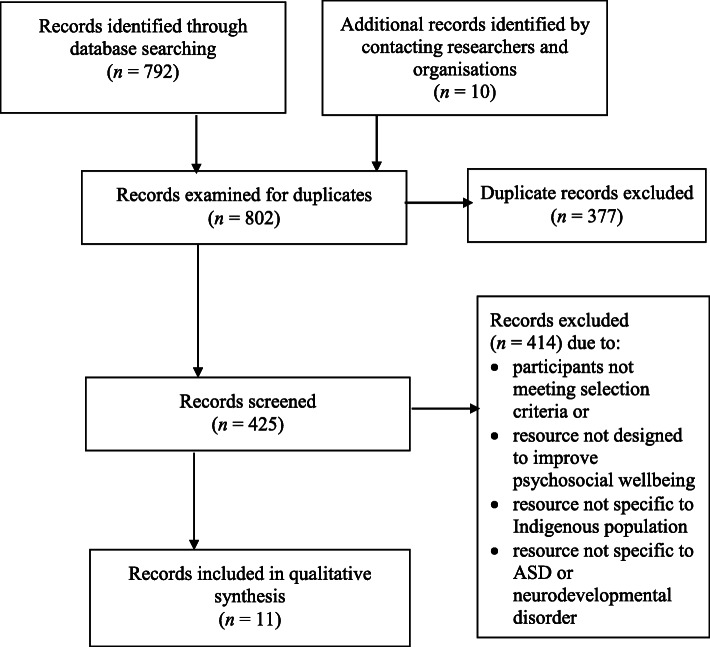
Flow diagram showing article selection process for the catalogue of psychosocial resources

Figure 2 illustrates the article selection process for the catalogue of psychosocial resources. Database searches retrieved a total of 792 articles, none of which were reviews, and 10 articles were identified by contacting researchers and organisations. Examining the 802 articles for duplicates resulted in the removal of 377 duplicates, leaving 425 articles to be screened. Examining the titles, abstracts and, where necessary, the full text of the remaining articles against the inclusion criteria resulted in 414 exclusions, leaving 11 relevant articles (see Table 2). Reasons for exclusion included the resource not addressing Indigenous people with a neurodevelopmental disorder or not aiming to improve psychosocial wellbeing. Specifically, one resource was described as Indigenous as it was a program developed for people living in a certain area but was excluded as it was not developed for Indigenous people [72].

**Table 2:** Psychosocial resources developed for Indigenous people with ASD characteristics, and/or their caregivers

Resource		Developers	Type	Targeted population	Registration requirements	Assistance required	Cost and accessibility	Evaluated?
1	Aboriginal & Torres Strait Islander Peoples psychoeducation and support	Positive Partnerships	Website offering online psychoeducation with downloadable resources.	Parents, teachers and community engaged with children with ASD	Webinar access and community programs	Community programs are run by Positive Partnerships staff	Free. Online resources easily accessible. Must register for community program.	Yes
2	Alert Program Study	Telethon Kids Institute, Communities of the Fitzroy Valley, and Bree Wagner (lead researcher)	A school-based intervention program delivered through school curriculum that teaches students about self-regulation and provides them with strategies to improve focus and emotion regulation.	The program is delivered by teachers to all students, with a particular research focus on outcomes for students with FASD	Initial research has finished recruitment	Initial training delivered to teachers by researchers, teachers continue to implement the curriculum in the Fitzroy Valley	Free. Currently delivered only in the rural area of Fitzroy Valley, Western Australia as this area is disproportionately affected by FASD.	Yes – see Article F in systematic review for pilot study outcomes
3	Autism Aboriginal Way	Community Facebook Group	Online group providing support for Aboriginal people that identify as Autistic	Caregivers of Aboriginal children and adolescents with a diagnosis of ASD	Users must request to join the group and complete a short survey	No	Free. Access requires a Facebook account and internet access.	No
4	Autism Spectrum Australia Cultural and Indigenous Support	Autism Spectrum Australia	Resource providing psychoeducation and support for caregivers.	Parents or carers of Indigenous children with ASD	No	No	Free. Access requires internet access.	No
5	Autism Teen Wellbeing website	Lead researchers Ian Shochet and Beth Saggers in collaboration with Autism CRC, Positive Partnerships, & Queensland University of Technology	Website providing psychoeducation, resources, and cultural considerations for building resilience among teenagers with ASD within a multi-level approach.	Using a multi-level approach highlighting parents, carers, teachers, support staff, schools, and the wider community as supports for teens with ASD	No	No	Free. Access requires internet access.	No
6	Be My Koorda Support Group	Community Facebook Group and Online Website	Online group for caregivers. Shares information and strategies, and holds face-to-face meetings in Koorda, Western Australia.	Parents or carers of Indigenous children with ASD and other disabilities	Users must request to join the social media group	No	Free. Access requires a Facebook account and/or internet access. Meetings restricted to Koorda, Western Australia.	No
7	Early Days Workshops for Aboriginal and Torres Strait Islander Families	Secretariat of National Aboriginal and Islander Child Care (SNAICC) and Early Days – Promoting Development of Young Children on the Autism Spectrum	Educational workshops (online and face-to-face) to build resilience and provide information about Autism and intervention approaches. Access to local services during assessment and after diagnosis.	Parents or carers of young children with ASD, up to 6 years of age	Registration required	Yes, face-to-face workshops are delivered by Early Days staff in each state and territory to compliment online workshops	Free. Online workshops available to all. Face-to-face workshops have to be organised with the Early Days Team in each state or territory.	Yes: by Aboriginal parent, child care worker, and SNAICC. Ongoing post-workshop parent evaluations.
8	Early Intervention: Indigenous Liaison Officer Program (EI ILO)	Autism Queensland	Resource increasing awareness of childhood disability and diagnosis, benefits of early intervention, and links to culturally relevant services.	Parents, carers, families and communities of Indigenous children with ASD	No	No	Free. Available to all seeking support and information. Requires internet access	No
9	Four Directions Autism website	Volunteer-run support group	Website providing psychoeducational resources and support for parents.	Parents of First Nations children with ASD	No	No	Free. Online resources easily accessible.	No
10	Paediatric Child Health and Education Services (PATCHES)	PATCHES	Diagnostic and neuropsychology assessments for FASD, ASD, and other developmental disorders; a range of therapy services; and outreach programs.	Uniting schools and families with clinicians to support those with FASD, ASD, or other learning or developmental difficulties who live in remote and regional areas	Registration required	Yes	Free. Available in key communities in Western Australia, Northern Territory, New South Wales, and Tasmania.	No
11	Takiwātanga Māori Autism Support Group	Community Facebook Group	Online support for whānau (family) who have a child with ‘Takiwātanga’ (Autism) through the diagnosis system.	Whānau (family) who have a child or family member with ‘Takiwātanga’ (Autism)	Users must request to join the social media group	No	Free. Access requires a Facebook account and internet access.	No

The majority of the 11 psychosocial resources identified through this review were developed in Australia (see Table 2; Resources 1–8 & 10), while Resource 9 was developed in Canada and Resource 11 was developed in New Zealand. Ten resources provide support for parents, families and carers (Resources 1, 3–11); however, only two resources were described as a resource for the Indigenous person with ASD and/or another neurodevelopmental disorder (Resources 2 & 10). Four resources support teachers (Resources 1, 2, 5, & 10) and three resources include support for communities (Resources 1, 5, & 8). Six of the resources (Resources 1, 4–6, 8, & 9) provide psychoeducation about ASD or other neurodevelopmental disorders, four resources (Resources 3, 4, 9, & 11) provide community and parent support, and three resources (Resources 2, 7, & 10) provide a service, in the form of an intervention, workshops, and therapy for people with ASD and/or another neurodevelopmental disorder and/or their caregivers. Only three resources (Resources 1, 2, & 7) had been evaluated to ensure their validity in providing positive outcomes.

All of the resources identified in this review are free to use, however six of the resources were developed and are run by organisations that rely on funding (Resource 1 – Positive Partnerships, Resource 2 – Telethon Kids Institute, Resource 4 – Autism Spectrum Australia, Resource 5 – The School Connectedness Project [Autism CRC, Positive Partnerships, and Queensland University of Technology], Resource 7 – SNAICC and Early Days, Resource 8 – Autism Queensland). Nine resources require internet access in order to engage with the resource platform or to participate in online workshops. Resources 1 and 7 use a combination of online and face-to-face workshops. Conducted by staff members of organisations such as Positive Partnerships, SNAICC, and Early Days, the workshops are run on an “as needed” basis and require contact with the program organisers. Resources 2 and 10 deliver services face-to-face (classroom intervention and therapy services) but only in specific communities. Resource 6 also conducts face-to-face support meetings for parents. Utilising face-to-face delivery limits accessibility for those living in rural and remote areas, or those living internationally. Resources 3, 6, and 11 are closed Facebook groups, and in order to participate, the user must have a Facebook account and must ask to be accepted into the group by the administrator. Resources 5 and 9 require only an internet connection. While the Four Directions website provides information specific to the country in which the resource was developed (Canada), the Autism Teen Wellbeing website discusses more universal concepts and approaches that could be implemented across the globe.

## Discussion

There is a demonstrated increased risk for Indigenous children, adolescents, and adults with ASD (and/or another neurodevelopmental disorder), and their caregivers, of experiencing psychosocial difficulties, however this systematic review revealed a paucity of literature (regardless of publishing status) relating to programs or resources promoting psychosocial wellbeing in this population. Despite the comprehensiveness of the systematic literature search, only seven articles published in peer-reviewed or grey literature were identified (four of which came from two separate studies), and only eleven resources were identified.

### Systematic review

Several trends can be identified in the articles reported in this systematic review. All articles described research conducted in North America or Australia and were published within the last 10 years, indicating that research with Indigenous people with ASD and/or another neurodevelopmental disorder is a recent focus of the literature, and suggesting that research of this nature is not being conducted world-wide. Further, all articles focused on supporting children, highlighting the lack of research across other stages of the life span. A strength of the literature identified in this review includes the acknowledgement of cultural factors in the research process, and the importance of collaborating with traditional Elders and knowledge keepers. This was achieved through the inclusion of Indigenous research methods in the study design (see Table 1; Articles C & D), the inclusion of Indigenous people in the team of evaluators who reviewed programs (Article E - G), and follow up interviews with participants to strengthen the accuracy of findings. Most articles (5 out of 7) described programs that culturally adapted existing programs to improve psychosocial wellbeing for people with ASD and/or another neurodevelopmental disorder (Articles A, B, & E - G). As such, the literature shows promise that psychosocial wellbeing can be realised through designing programs for Indigenous people, or through careful cultural adaptation of an existing evidence-based program.

Across all articles, a number of factors outlined the gaps in this area of research. Only two studies reported quantitative data that was gathered using validated scales (Bettag [65] Article A; Wagner et al. [70] Article F). All other articles reported qualitative outcomes, analysing data from a combination of sources (e.g., interviews, field notes, video recordings). Four articles (Articles B - E) analysed small samples, ranging from 2 to 5 participants that yielded a wealth of qualitative information about participants and their participation outcomes. Bettag (Article A) [65] and Wagner et al. (Articles F & G) [70, 71] were the only studies with larger (although still small) sample sizes. At the time of this review, no research had conducted a randomised control trial to evaluate programs or workshops that aim to improve psychosocial wellbeing in this population. This is understandable given the ethical and logistical challenges of conducting research with Indigenous populations, including political and financial intricacies that require successful navigation when engaging and collaborating with Indigenous communities in intervention studies, complexities sourcing participants who meet inclusion criteria due to lower diagnosis rates, difficulties building trust with participants due to Indigenous communities’ adverse experience historically of researchers, and the increased time required to complete an intervention study rather than a descriptive study which can be further augmented in communities with high mobility, all of which can reduce sample size (and also impede the creation of a control group), increase attrition, and reduce the likelihood of non-significant results for which there are decreased publishing options.

In summary, the systematic review revealed a small pool of literature regarding programs that promote psychosocial wellbeing for Indigenous people with characteristics or a diagnosis of ASD and/or other neurodevelopmental disorders, and/or the psychosocial wellbeing of their caregivers. The available literature revealed that culturally adapting existing evidence-based programs showed as much promise as designing new programs for this population. Given the complexities of designing and evaluating new programs, the careful and appropriate cultural adaption of existing evidence-based programs would increase feasibility of ongoing research and not compromise outcomes.

### Psychosocial resources

Of the eleven resources identified in this review, only three had undergone evaluation. While the existence of the identified resources is promising, it is unknown whether many of them are sufficiently supporting psychosocial wellbeing in this population. Nine resources required internet access in order to engage with the online materials or workshops. For Indigenous people in Australia, access to the internet has increased significantly over the past 12 years. Daly [73] found that non-Indigenous people were three times more likely to have access to technology and the internet than Indigenous people identified by the 2001 Australian Census of Population and Housing. The more recent 2014–15 National Aboriginal and Torres Strait Islander Social Survey [74] showed that 56% of Indigenous people in remote, and 64% in non-remote areas had daily access to the internet. A recent study completed with the Ngarrindjeri people [75] found that the participants considered the internet to be an important source of information, second only to information they could source from family members. The Ngarrindjeri people’s recognition of the internet as a source of information supports its use for programs and resources that promote psychosocial wellbeing among this population, and as a space where Indigenous people with characteristics or a diagnosis of ASD and/or another neurodevelopmental disorder and their caregivers can share their experiences.

The findings of the current review identified only a small collection of literature researching programs or resources promoting psychosocial wellbeing for Indigenous people with ASD and/or other neurodevelopmental disorders. There is an extensive range of psychosocial resources that have been successfully developed across the developmental stages that could be adapted with careful community based participatory research (CBPR) practices. The CBPR approach acknowledges the community as an equal partner in all aspects of the research [76]. Our research team recently conducted a strength-based workshop for Indigenous community workers who work directly with Indigenous populations in Bourke which is a town in a remote area of Northern New South Wales in Australia. The workshop in Bourke was part of community based participatory research that aimed to develop a sustainable program to promote wellbeing and support inclusion and connectedness for adolescents with ASD, their families, and the wider community. Prior to the workshop, key stakeholders from the community were consulted about the nature of a program that would best meet the needs of the community, which led to the development of the Resourceful Adolescent Parent and Caregiver Program (RAP-PC) [77]. The aims of RAP-PC are: (1) to boost the self-efficacy of parents and caregivers, (2) to help parents and caregivers to manage their stress so that they can react calmly and be less over-reactive to their adolescents, (3) to help boost their adolescent’s self-esteem, sense of belonging, and resilience, and (4) to promote positive family relationships and to reduce and manage conflict in the family system.

The three-day workshop trained community workers in the RAP-PC program so that they could then implement the program with parents and families in their communities. Throughout the workshop, the community workers were consulted about the relevance of the program for their communities. Congruent with the findings described in this paper, during the training workshop the community workers described the lack of awareness, services, and resources specific to Indigenous people with ASD, and described the many challenges of obtaining a diagnosis and support. All community workers who participated in the workshop were seeking programs, workshops or resources to support Indigenous people with ASD in their communities, further emphasising the need for such programs.

### Strengths and limitations

A strength of this study is its thorough review of academic databases and sources of published and grey academic literature. The search strategies were developed and refined through collaboration between the research team and a research librarian to ensure the appropriateness and efficacy of their scope. Publications in all languages and from all databases were eligible for inclusion. The comprehensive and exhaustive search included 28 databases for research evaluating programs aiming to promote psychosocial wellbeing, and 25 databases for resources designed to promote psychosocial wellbeing. Additional contact with researchers in the field revealed no further research or resources (completed or underway) that met the eligibility criteria, supporting the comprehensiveness of the search process.

Another strength of this study includes the congruence of the findings, with reports from a small sector of the Australian Indigenous community adding weight to its findings. The RAP-PC workshop in Bourke demonstrated the importance of a strength-based approach when working with Indigenous people as partners in community development projects and facilitated incorporation of the community’s feedback in future recommendations. Finally, this review has compiled a table of existing resources available for Indigenous people with ASD and/or other neurodevelopmental disorders, and their caregivers. While only a small number of resources were identified in this review, it is possible that resources unavailable in the public domain may be being used in Indigenous communities.

This review has identified that this is an under-researched area in a population of people who need to overcome a number of barriers to access support. The systematic review identified only 7 articles of moderate to low methodological quality, and the diversity and paucity of outcomes reported precluded the calculation of an overall effect of the interventions described [78]. In addition, the majority of articles (*n* = 4, 57%) used a qualitative design which may be explained partly by the ethical and logistical challenges of conducting research with Indigenous populations; and in turn, can reduce sample size, impede the creation of a control group, increase attrition, and reduce the likelihood of non-significant results for which there are decreased publishing options. Further, only 11 resources providing psychosocial wellbeing supports were found. The scarcity of published research and resources developed to promote psychosocial wellbeing in this population, and the lower methodological rigour of research studies does not appear to have improved over the past 17 years: a literature review of Indigenous health research conducted world-wide and published between 1987 and 2003 found that only 7% of articles reported outcomes from programs or interventions, and attributed this paucity to the increased time required to complete an intervention study rather than a descriptive study deterring the research from being conducted; the political and financial challenges that needed to be successfully navigated when engaging and collaborating with Indigenous communities in an intervention study; lack of funding for multi-level, community-wide interventions; and decreased publishing options for intervention studies with non-significant results [79]. The lack of studies overall, the absence of any studies of high methodological quality, and the scarcity of resources identified can be seen as a limitation of the current study, but also emphasises the need for future research to develop and evaluate programs that promote psychosocial wellbeing for Indigenous people with characteristics or a diagnosis of ASD and/or other neurodevelopmental disorders, as well as that of their caregivers.

The paucity of research suggests that Indigenous people with characteristics or a diagnosis of ASD and/or other neurodevelopmental disorders may be deprived of support at critical points of development or may resort to using supports that lack cultural awareness or competency, which may impact on their overall psychosocial wellbeing. However, if future research can leverage off existing evidence-based programs that improve psychosocial wellbeing and adapt them to the needs of Indigenous communities through ongoing collaboration, challenges relating to feasibility and impact can be minimised. As intervention-based research is a key component in creating change [79], it should be further pursued to realise positive improvements in health outcomes for this population.

Future research should focus on collaboratively developing culturally accepted programs that promote psychosocial wellbeing for Indigenous children, adolescents, and adults with characteristics or a diagnosis of ASD and/or other neurodevelopmental disorders, and/or the psychosocial wellbeing of their caregivers. Adopting a CBPR approach in this research may help to minimise obstacles that inhibit Indigenous people’s engagement in and utilisation of such programs. In Australia, major barriers to participation in research include the difficulties in accessing a diagnosis and difficulties in building trust with researchers [80]. Researchers can build trust and accountability by adhering to the guidelines for ethical research with Indigenous communities and investing the time to build reciprocal relationships with Indigenous people [81–83].

Additional research is needed internationally to develop and evaluate methods for increasing access to assessment and diagnosis, possibly by implementing diagnostic pathways that are accessible [18, 84], culturally sensitive to the historical experiences of Indigenous people [7, 85], and that cater for the effect of culture on symptom expression [11, 86]. Doing so will help to inform prevalence rates of ASD in Indigenous populations. The use of culturally sensitive tools for assessment and diagnosis is crucial so that adequate programs, psychoeducation, and support are made available to the Indigenous population [87].

Technology, such as the internet and video conferencing, has been successfully used for other mental health programs specific to Indigenous populations [84, 88–90]. As such, the internet provides a promising platform for future development of programs promoting psychosocial wellbeing and may go some way to addressing the need for accessible resources that can support people with characteristics or a diagnosis of ASD and/or other neurodevelopmental disorders, as well as their caregivers.

## Conclusions

This study conducted a systematic review of programs promoting psychosocial wellbeing for Indigenous people with characteristics or a diagnosis of ASD and/or other neurodevelopmental disorders, and the psychosocial wellbeing of their caregivers, and collated and reviewed resources designed to support this population. The results revealed a paucity of published and unpublished literature and few resources, but there were important exemplars. It is encouraging to note that through careful culturally appropriate adaptation processes, programs can be successfully adapted and implemented, making valuable resources available to Indigenous populations. What is required therefore is the recognition of the importance of doing this research, and of providing resources to conduct this research. This review has highlighted the urgent need for this research, and we express the hope that more government resources can be dedicated to support research for Indigenous and First Nations people across the lifespan with ASD and/or other neurodevelopmental disorders.

## Data Availability

Not applicable.

## Appendix 1

**Table 3:** Simplified Search Strings used for Systematic Review of Psychosocial Programs

**Simplified 1**	
(Indigenous OR Aborigin* OR Maori OR Inuit* OR “First Nation*” OR “Native American*” OR Sami) AND (Autis* OR Asperger* OR “Neurodevelopmental disorder*” OR “Intellectual disabilit*” OR “Learning Disabilit*”) AND (Therap* OR Treatment* OR Intervention* OR Support* OR Program* OR Resource) AND (“Mental health” OR “Psychological health” OR Connectedness OR Wellbeing OR Psychosocial OR Resilience)	
**Simplified 2**	
(Indigenous OR Aborigin* OR “Native American*” OR Sami) AND (Autis* OR “Intellectual disabilit*”) AND (Support* OR Program* OR Resource) AND (Resilience OR Wellbeing OR “Mental health”)	

**Table 4:** Search String Breakdown

Population	Condition	Intervention/Supports	Outcome
**Simplified 1**			
Indigenous OR Aborigin* OR Maori OR Inuit* OR “First Nation*” OR “Native American*” OR Sami	Autis* OR Asperger* OR “Neurodevelopmental disorder*” OR “Intellectual disabilit*” OR “Learning Disabilit*”	Therap* OR Treatment* OR Intervention* OR Support* OR Program* OR Resource	“Mental health” OR “Psychological health” OR Connectedness OR Wellbeing OR Psychosocial OR Resilience
**Simplified 2**			
Indigenous OR Aborigin* OR “Native American*” OR Sami	Autis* OR “Intellectual disabilit*”	Support* OR Program* OR Resource	Resilience OR Wellbeing OR “Mental health”

## Appendix 2

**Table 5:** Standard Search String for Systematic Review of Psychosocial Programs

Standard Search String	
(Indigenous OR Aborigin* OR “Torres Strait” OR Maori OR Inuit* OR Eskimo* OR Meti OR “First Nation” OR “First people” OR “Native American*” OR “American Indian*” OR “North American Native*” OR “Alaska* Native” OR “Native Alaska*” OR Amerind* OR Sami OR Saami OR Laplander* OR Lapp* OR “Native Alaskian*” OR Kalaallit* OR Inupiat* OR Aleut* OR “Pacific Island*” OR “Native Hawaiian” OR “Hawaiian native*” OR Yupik OR Athabaska* OR “Native Polynesia”) AND (Autis* OR Asperger* OR “Neurodevelopmental disorder” OR “Intellectual disability*” OR “Intellectual development* disorder” OR “Developmental delay” OR “Communicati* disorder” OR “Language disorder” OR “Speech sound disorder” OR “Childhood-onset fluency disorder” OR “Pragmatic language impairment” OR “Attention-deficit/hyperactivity disorder” OR ADHD OR “Attention deficit disorder” OR “specific learning disorder” OR “Motor disorder” OR “developmental coordination disorder” OR “Stereotypic movement disorder” OR “tic disorder” OR Tourette* OR “Autistic disorder” OR “Pervasive developmental disorder not otherwise specified” OR “PDD-NOS” OR “Childhood disintegrative disorder” OR “Semantic-pragmatic disorder” OR “Oppositional Defiant Disorder” OR “Disruptive Behavio* disorder” OR “Attention Deficit Hyperactivity Disorder” OR “Attention Deficit-Hyperactivity Disorder” OR “Hyperkinetic Syndrome” OR ADDH OR “Development* Disorder” OR “Development* disability” OR “Kanner* Syndrome” OR “Phonological Disorder” OR “Development* Deviation” OR “Mental retardation” OR “Developmental Academic disorder” OR “Learning Disabilit*” OR “developmental dyscalculia” OR Dyslexia OR “reading disorder” OR “reading disability” OR “motor skills disorder” OR mutism OR “Kussmaul* aphasia” OR “Neuro atypical”) AND (Therap* OR Treatment* OR Intervention* OR Support* OR Program* OR Service* OR Project* OR Assistance OR Tool* OR Training OR Resource OR Application OR App OR Counselling OR Counseling) AND (“Mental health” OR “Psychological health” OR Connectedness OR Wellbeing OR Well-being OR Happiness OR Psychosocial OR “Social skills” OR “Social functioning” OR Belonging OR Resilience OR Interpersonal OR self-esteem OR “self esteem” OR “Community connection” OR “emotional development” OR “social development” OR “spiritual development” OR “spiritual health” OR “emotional health” OR “social health” OR “spiritual connection” OR spirituality)	

**Table 6:** Search Strings Breakdown

Population	Condition	Intervention/Supports	Outcome
**Standard**			
Indigenous OR Aborigin* OR “Torres Strait” OR Maori OR Māori OR Inuit* OR Eskimo* OR Meti OR Méti OR “First Nation” OR “First people*” OR “Native American*” OR “American Indian*” OR “North American Native*” OR “Alaska* Native” OR “Native Alaska*” OR Amerind* OR Sami OR Saami OR Sámi OR Laplander* OR Lapp* OR “Native Alaskian*” OR Kalaallit* OR Inupiat* OR Aleut* OR “Pacific Island*” OR “Native Hawaiian*” OR “Hawaiian native*” OR Yupik OR Athabaska* OR “Native Polynesia*” excl. Native, tribal, tribe, OR Indígena* OR Aborígene* OR Índio* OR Indio* OR autochtone OR Urfolk OR Alkuasukas OR Inhemsk OR Indigene OR Aborigène OR лопари́ OR туземец* OR абориген* OR “Indians of north America”	Autis* OR Asperger* OR “Neurodevelopmental disorder*” OR “Intellectual disabilit*” OR “Intellectual development* disorder” OR “Developmental delay” OR “Communicati* disorder” OR “Language disorder” OR “Speech sound disorder” OR “Childhood-onset fluency disorder” OR “Pragmatic language impairment” OR “Attention-deficit/hyperactivity disorder” OR ADHD OR “Attention deficit disorder” OR “Specific learning disorder” OR “Motor disorder” OR “Developmental coordination disorder” OR “Stereotypic movement disorder” OR “Tic disorder” OR Tourette* OR “Autistic Disorder” OR “Pervasive developmental disorder not otherwise specified” OR “PDD-NOS” OR “Childhood disintegrative disorder” OR “Semantic-pragmatic disorder” OR “Oppositional Defiant Disorder” OR “Disruptive Behavio* Disorder” OR “Attention Deficit Hyperactivity Disorder” OR “Attention Deficit-Hyperactivity Disorder” OR “Hyperkinetic Syndrome” OR ADDH OR “Development* Disorder” OR “Development* disability” OR “Kanner* Syndrome” OR “Phonological Disorder” OR “Development* Deviation” OR “Mental Retardation” OR “Developmental Academic Disorder” OR “Learning Disabilit*” OR “Developmental Dyscalculia” OR Dyslexia OR “Reading Disorder” OR “Reading disability” OR “Motor Skills Disorder” OR Mutism OR “Kussmaul* Aphasia” OR “Neuro atypical” excl. ADD, SCD	Therap* OR Treatment* OR Intervention* OR Support* OR Program* OR Service* OR Project* OR Assistance OR Tool* OR Training OR Resource OR Application OR App OR Counselling OR Counseling	“Mental health” OR “Psychological health” OR Connectedness OR Wellbeing OR Well-being OR Happiness OR Psychosocial OR “Social skills” OR “Social functioning” OR Belonging OR Resilience OR Interpersonal OR self-esteem OR “self esteem” OR “Community connection” OR “emotional development” OR “social development” OR “spiritual development” OR “spiritual health” OR “emotional health” OR “social health” OR “spiritual connection” OR spirituality

## Appendix 3

The 28 electronic databases searched when conducting the systematic review of psychosocial programs included:

1. Academic Search Elite.
2. Allied and Complimentary Medicines Database (AMED).
3. Australian Indigenous HealthInfoNet.
4. Bielefeld Academic Search Engine (BASE).
5. CINAHL (on EBSCOHost).
6. Cochrane Library (on Wiley).
7. Circumpolar Health Bibliographic Database (on Canadian Institutes of Health Research).
8. EMBASE (on Elsevier).
9. ERIC, Indigenous Studies Database (on the Queensland University of Technology Indigenous Studies Research Network).
10. Indigenous Studies Portal.
11. Informit.
12. JSTOR.
13. Learning Ground.
14. Proquest Health and Medical Collection.
15. Proquest Psychology.
16. PsycARTICLES.
17. PsycINFO.
18. PubPsych (on Proquest).
19. PubMed (on NCBI).
20. ScienceDirect (on Elsevier).
21. Scopus.
22. Social Science Research Network (on Elsevier).
23. Social Services Abstracts (on Proquest).
24. Social Work Abstracts.
25. Web of Science (on Web of Knowledge).
26. Open Grey.
27. Open Access Theses and Dissertations.
28. ProQuest Dissertations and Theses Open.

## Appendix 4

The 25 electronic databases searched when compiling the catalogue of psychosocial resources included:

1. Academic Search Elite (on EBSCOHost).
2. Allied and Complementary Medicine Database (on EBSCOHost).
3. Australian Indigenous HealthInfoNet.
4. CINAHL (on EBSCOHost).
5. Cochrane Library (on Wiley).
6. ERIC (on EBSCOHost).
7. Informit.
8. Learning Ground.
9. Medline (on EBSCOHost).
10. Proquest Psychology.
11. PsycArticles (on EBSCOHost).
12. PsycINFO (on EBSCOHost).
13. PubPsych (on Proquest).
14. PubMed (on NCBI).
15. SAGE.
16. Science Direct.
17. Scopus.
18. Social Services Abstracts (on Proquest).
19. Sociological Abstracts.
20. Springer Link.
21. Web of Science (on Web of Knowledge).
22. Google Scholar.
23. Microsoft Academic.
24. Advanced Google search engine.
25. Quick Find from the Queensland University of Technology Library.

## Abbreviations

ABA: Adaptive Behavioural Analysis
ADHD: Attention-Deficit/Hyperactivity Disorder
ASD: Autism Spectrum Disorder
Autism CRC: Australian Cooperative Research Centre for Living with Autism
CBPR: Community Based Participatory Research
DSM 5: Diagnostic and Statistical Manual of Mental Disorders (5th ed.)
EI ILO: Early Intervention: Indigenous Liaison Officer Program
FASD: Fetal Alcohol Spectrum Disorder
MMAT: Mixed Methods Assessment Tool
PATCHES: Paediatric Child Health and Education Services
PDD-NOS: Pervasive Developmental Disorder not Otherwise Specified
PRISMA: Preferred Reporting Items for Systematic Review and Meta-analysis
RAP-PC: Resourceful Adolescent Parent and Caregiver Program
SNAICC: Secretariat of National Aboriginal and Islander Child Care

## References

[1] United Nations Forum on Indigenous Issues (2007). Indigenous peoples, Indigenous voices.

[2] Elsabbagh M, Divan G, Koh YJ, Kim YS, Kauchali S, Marcín C, Montiel-Nava C, Patel V, Paula CS, Wang C (2012). Global prevalence of autism and other pervasive developmental disorders. Autism Res.

[3] Hansen SN, Schendel DE, Parner ET (2015). Explaining the increase in the prevalence of autism spectrum disorders: the proportion attributable to changes in reporting practices. JAMA Pediatr.

[4] Idring S, Lundberg M, Sturm H, Dalman C, Gumpert C, Rai D, Lee B, Magnusson C (2015). Changes in prevalence of autism spectrum disorders in 2001–2011: findings from the Stockholm youth cohort. J Autism Dev Disord.

[5] Matson JL, Kozlowski AM (2011). The increasing prevalence of autism spectrum disorders. Res Autism Spectr Disord.

[6] Australian Institute of Health and Welfare (2016). Australian Burden of Disease Study 2011: Impact and causes of illness and death in Aboriginal and Torres Strait Islander people. Australian Burden of Disease Study Series No. 6. Cat. No. BOD 7.

[7] Bennett M, Hodgson V (2017). The missing voices of indigenous Australians with autism in research. Autism..

[8] Mitchell D, Mitchell D (2005). Introduction: sixteen propositions on the contexts of inclusive education. Contextualizing inclusive education: evaluating old and new international perspectives.

[9] Avery S (2018). Culture is inclusion: a narrative of aboriginal and Torres Strait islander people with disability. First Peoples Disability Network Australia.

[10] American Psychiatric Association (2013). Diagnostic and statistical manual of mental disorders: DSM-5.

[11] Roy M, Balaratnasingam S (2010). Missed diagnosis of autism in an Australian indigenous psychiatric population. Australas Psychiatry..

[12] Anthony JH (2010). Towards inclusion: influences of culture and internationalisation on personhood,educational access, policy and provision for students with autism in Ghana. PhD [dissertation].

[13] Bernier R, Mao A, Yen J (2010). Psychopathology, families, and culture: autism. Child Adolesc Psychiatr Clin N Am.

[14] Ouellette-Kuntz H, Coo H, Yu CT, Chudley AE, Noonan A, Breitenbach M, Ramji N, Prosick T, Bedard A, Holden JJA (2006). Prevalence of pervasive developmental disorders in two Canadian provinces. J Policy Pract Intellect Disabil.

[15] Leonard H, Glasson E, Nassar N, Whitehouse A, Bebbington A, Bourke J, Jacoby P, Dixon G, Malacova E, Bower C, Stanley F (2011). Autism and intellectual disability are differentially related to sociodemographic background at birth. PLoS One.

[16] Mandell DS, Novak MM, Zubritsky CD (2005). Factors associated with age of diagnosis among children with autism spectrum disorders. Pediatrics..

[17] Burstyn I, Sithole F, Zwaigenbaum L (2010). Autism spectrum disorders, maternal characteristics and obstetric complications among singletons born in Alberta, Canada. Chronic Dis Can.

[18] Wilson K, Watson L (2011). Autism spectrum disorder in Australian indigenous families: issues of diagnosis, support and funding. Aborig Isl Health Work J.

[19] Eley D, Young L, Hunter K, Baker P, Hunter E, Hannah D (2007). Perceptions of mental health service delivery among staff and indigenous consumers: it's still about communication. Australas Psychiatry.

[20] Ben Itzchak E, Zachor DA (2011). Who benefits from early intervention in autism spectrum disorders?. Res Autism Spectr Disord.

[21] Howlin P (1997). Prognosis in autism: do specialist treatments affect long-term outcome?. Eur Child Adolesc Psychiatry.

[22] Reichow B (2012). Overview of meta-analyses on early intensive behavioral intervention for young children with autism spectrum disorders. J Autism Dev Disord.

[23] Ching H, Pringsheim T (2012). Aripiprazole for autism spectrum disorders (ASD). Cochrane Database Syst Rev.

[24] Jesner OS, Aref-Adib M, Coren E (2007). Risperidone for autism spectrum disorder. Cochrane Database Syst Rev.

[25] Matson JL, Horovitz M (2010). Stability of autism spectrum disorders symptoms over time. J Dev Phys Disabil.

[26] Matson JL, Cervantes PE, Peters WJ (2016). Autism spectrum disorders: management over the lifespan. Expert Rev Neurother.

[27] Mannion A, Brahm M, Leader G (2014). Comorbid psychopathology in autism spectrum disorder. J Autism Dev Disord.

[28] Marsh A, Spagnol V, Grove R, Eapen V (2017). Transition to school for children with autism spectrum disorder: a systematic review. World J Psychiatry.

[29] Zablotsky B, Bradshaw CP, Anderson CM, Law P (2014). Risk factors for bullying among children with autism spectrum disorders. Autism..

[30] Bauminger N, Shulman C, Agam G (2003). Peer interaction and loneliness in high-functioning children with autism. J Autism Dev Disord.

[31] Humphrey N, Symes W (2010). Responses to bullying and use of social support among pupils with autism spectrum disorders (ASDs) in mainstream schools: a qualitative study. J Res Spec Educ Needs.

[32] White S, Roberson-Nay R (2009). Anxiety, social deficits, and loneliness in youth with autism spectrum disorders. J Autism Dev Disord.

[33] Mazurek M, Kanne S (2010). Friendship and internalizing symptoms among children and adolescents with ASD. J Autism Dev Disord.

[34] Mayes SD, Calhoun SL, Murray MJ, Zahid J (2011). Variables associated with anxiety and depression in children with autism. J Dev Phys Disabil.

[35] McPheeters ML, Davis A, Navarre JR, Scott TA (2011). Family report of ASD concomitant with depression or anxiety among US children. J Autism Dev Disord.

[36] Mazefsky CA, White SW (2014). Emotion regulation: concepts & practice in autism spectrum disorder. Child Adolesc Psychiatr Clin N Am.

[37] Samson AC, Huber O, Gross JJ (2012). Emotion regulation in Asperger's syndrome and high-functioning autism. Emotion..

[38] Jahromi LB, Meek SE, Ober-Reynolds S (2012). Emotion regulation in the context of frustration in children with high functioning autism and their typical peers. J Child Psychol Psychiatry.

[39] Konstantareas MM, Stewart K (2006). Affect regulation and temperament in children with autism spectrum disorder. J Autism Dev Disord.

[40] Dalton KM, Nacewicz BM, Johnstone T, Schaefer HS, Gernsbacher MA, Goldsmith HH, Alexander AL, Davidson RJ (2005). Gaze fixation and the neural circuitry of face processing in autism. Nat Neurosci.

[41] Samson AC, Hardan AY, Podell RW, Phillips JM, Gross JJ (2015). Emotion regulation in children and adolescents with autism spectrum disorder. Autism Res.

[42] Bruggink A, Huisman S, Vuijk R, Kraaij V, Garnefski N (2016). Cognitive emotion regulation, anxiety and depression in adults with autism spectrum disorder. Res Autism Spectr Disord.

[43] Tantam D (2014). Adults with ASD. Curr Dev Disord Rep.

[44] Cai RY, Richdale AL (2016). Educational experiences and needs of higher education students with autism spectrum disorder. J Autism Dev Disord.

[45] Mojtabai R, Stuart EA, Hwang I, Eaton WW, Sampson N, Kessler RC (2015). Long-term effects of mental disorders on educational attainment in the National Comorbidity Survey ten-year follow-up. Soc Psychiatry Psychiatr Epidemiol.

[46] Hendricks D (2010). Employment and adults with autism spectrum disorders: challenges and strategies for success. J Vocat Rehabil.

[47] Taylor JL, Henninger NA, Mailick MR (2015). Longitudinal patterns of employment and postsecondary education for adults with autism and average-range IQ. Autism..

[48] Strunz S, Schermuck C, Ballerstein S, Ahlers CJ, Dziobek I, Roepke S (2017). Romantic relationships and relationship satisfaction among adults with Asperger syndrome and high-functioning autism. J Clin Psychol.

[49] Bronfenbrenner U (1979). The ecology of human development experiments by nature and design.

[50] Meadan H, Halle JW, Ebata AT (2010). Families with children who have autism spectrum disorders: stress and support. Except Child.

[51] Anagnostou E, Zwaigenbaum L, Szatmari P, Fombonne E, Fernandez BA, Woodbury-Smith M, Brian J, Bryson S, Smith IM, Drmic I (2014). Autism spectrum disorder: advances in evidence-based practice. CMAJ..

[52] Ingersoll B, Hambrick DZ (2011). The relationship between the broader autism phenotype, child severity, and stress and depression in parents of children with autism spectrum disorders. Res Autism Spectr Disord.

[53] Da Paz NS, Wallander JL (2017). Interventions that target improvements in mental health for parents of children with autism spectrum disorders: a narrative review. Clin Psychol Rev.

[54] Mackay BA, Shochet IM, Orr JA (2017). A pilot randomised controlled trial of a school-based resilience intervention to prevent depressive symptoms for young adolescents with autism spectrum disorder: a mixed methods analysis. J Autism Dev Disord.

[55] McKenzie Smith M, Pinto Pereira S, Chan L, Rose C, Shafran R (2018). Impact of well-being interventions for siblings of children and young people with a chronic physical or mental health condition: a systematic review and meta-analysis. Clin Child Fam Psychol Rev.

[56] Mandell D, Walrath C, Manteuffel B, Sgro G, Pinto-Martin J (2005). Characteristics of children with autistic spectrum disorders served in comprehensive community-based mental health settings. J Autism Dev Disord.

[57] Able H, Sreckovic MA, Schultz TR, Garwood JD, Sherman J (2015). Views from the trenches: teacher and student supports needed for full inclusion of students with ASD. Teach Educ Spec Educ.

[58] Gates JA, Kang E, Lerner MD (2017). Efficacy of group social skills interventions for youth with autism spectrum disorder: a systematic review and meta-analysis. Clin Psychol Rev.

[59] Herrman HS, Saxena S, Moodie R (2005). Promoting mental health: concepts, emerging evidence, practice. A WHO report in collaboration with the Victorian Health Promotion Foundation and the University of Melbourne.

[60] Liberati A, Altman DG, Tetzlaff J, Mulrow C, Gøtzsche PC, Ioannidis JPA, Clarke M, Devereaux PJ, Kleijnen J, Moher D (2009). The PRISMA statement for reporting systematic reviews and meta-analyses of studies that evaluate healthcare interventions: explanation and elaboration. BMJ..

[61] Hong QN, Fàbregues S, Bartlett G, Boardman F, Cargo M, Dagenais P, Gagnon M-P, Griffiths F, Nicolau B, O'Cathain A (2018). The mixed methods appraisal tool (MMAT) version 2018 for information professionals and researchers. Educ Inf.

[62] Pace R, Pluye P, Bartlett G, Macaulay AC, Salsberg J, Jagosh J, Seller R (2012). Testing the reliability and efficiency of the pilot mixed methods appraisal tool (MMAT) for systematic mixed studies review. Int J Nurs Stud.

[63] Pluye P, Gagnon M-P, Griffiths F, Johnson-Lafleur J (2009). A scoring system for appraising mixed methods research, and concomitantly appraising qualitative, quantitative and mixed methods primary studies in mixed studies reviews. Int J Nurs Stud.

[64] Souto RQ, Khanassov V, Hong QN, Bush PL, Vedel I, Pluye P (2015). Systematic mixed studies reviews: updating results on the reliability and efficiency of the mixed methods appraisal tool. Int J Nurs Stud.

[65] Bettag D (2016). Analysis of the adaptation of the responsive teaching paradigm to serve predominantly native Hawaiian communities: a framework for guiding culturally appropriate, family-centered, relationship-based early childhood services. PhD [dissertation].

[66] Keightley M, Agnihotri S, Subramaniapillai S, Gray J, Keresztesi J, Colantonio A, Polatajko HJ, Cameron D, Wiseman-Hakes C (2018). Investigating a theatre-based intervention for indigenous youth with fetal alcohol spectrum disorder: exploration d’une intervention basée Sur le théâtre auprès de jeunes Autochtones atteints du syndrome d’alcoolisme fœtal. Can J Occup Ther.

[67] Lindblom A (2017). ‘It gives them a place to be proud’– music and social inclusion. Two diverse cases of young first nations people diagnosed with autism in British Columbia, Canada. Psychol Music.

[68] Lindblom A (2017). Exploring autism and music interventions through a first nations lens. AlterNative..

[69] Najera B (2012). ABA in native American homes: a culturally responsive training for paraprofessionals. PhD [dissertation].

[70] Wagner B, Olson HC, Symons M, Mazzucchelli TG, Jirikowic T, Latimer J, Watkins R, Cross D, Boulton J, Wright E (2019). Improving self-regulation and executive functioning skills in primary school children in a remote Australian aboriginal community: a pilot study of the alert program®. Aust J Educ.

[71] Wagner B, Cross D, Adams E, Symons M, Mazzucchelli TG, Watkins R, Wright E, Latimer J, Carapetis J, Boulton J. RE-AIM evaluation of a teacher-delivered programme to improve the self-regulation of children attending Australian aboriginal community primary schools. Emot Behav Diffic. 2019:1–17.

[72] Karanth P, Shaista S, Srikanth N (2010). Efficacy of communication DEALL - an indigenous early intervention program for children with autism spectrum disorders. Indian J Pediatr.

[73] Daly A (2006). Bridging the digital divide: the role of community online access centres in indigenous communities.

[74] Australian Bureau of Statistics (2016). National Aboriginal and Torres Strait islander social survey, Australia, 2014–15.

[75] Du JT, Haines J, Sun VQ, Partridge H, Ma D (2015). Understanding indigenous people's information practices and internet use: a Ngarrindjeri perspective.

[76] Ahmed SM, Palermo A-GS (2010). Community engagement in research: frameworks for education and peer review. Am J Public Health.

[77] Shochet IM, Wurfl AM (2018). Resourceful adolescent parent and caregivers program (RAP-PC).

[78] Higgins JPT, Green S (2011). Cochrane handbook for systematic reviews of interventions version 5.1.0.

[79] Sanson-Fisher RW, Campbell EM, Perkins JJ, Blunden SV, Davis BB (2006). Indigenous health research: a critical review of outputs over time. Med J Aust.

[80] Di Pietro N, Illes J (2016). Closing gaps: strength-based approaches to research with aboriginal children with neurodevelopmental disorders. Neuroethics..

[81] Australian Institute of Aboriginal and Torres Strait Islander Studies (2012). Guidelines for ethical research in Australian Indigenous studies.

[82] Canadian Institutes of Health Research, Natural Sciences and Engineering Research Council of Canada, and Social Sciences and Humanities Research Council of Canada: Research involving the First Nations, Inuit and Metis peoples of Canada. 2010. https://cihr-irsc.gc.ca/e/29339.html. Accessed 3 Mar 2019.

[83] Council NHaMR: Ethical conduct in research with aboriginal and Torres Strait islander peoples and communities: guidelines for research and stakeholders. 2018. https://www.nhmrc.gov.au/research-policy/ethics/ethical-guidelines-research-aboriginal-and-torres-strait-islander-peoples. Accessed 3 Mar 2019.

[84] Savin D, Garry MT, Zuccaro P, Novins D (2006). Telepsychiatry for treating rural American Indian youth. J Am Acad Child Adolesc Psychiatry.

[85] Hollinsworth D (2013). Decolonizing indigenous disability in Australia. Disabil Soc.

[86] Lindblom A (2014). Under-detection of autism among first nations children in British Columbia, Canada. Disabil Soc.

[87] Harris B, Barton EE, Albert C (2014). Evaluating autism diagnostic and screening tools for cultural and linguistic responsiveness. J Autism Dev Disord.

[88] Dingwall KM, Puszka S, Sweet M, Mills PPJR, Nagel T (2015). Evaluation of a culturally adapted training course in indigenous e-mental health. Australas Psychiatry..

[89] Dingwall KM, Puszka S, Sweet M, Nagel T (2015). ‘Like drawing into sand’: acceptability, feasibility, and appropriateness of a new e-mental health resource for service providers working with aboriginal and Torres Strait islander people. Aust Psychol.

[90] Povey J, Mills PPJR, Dingwall KM, Lowell A, Singer J, Rotumah D, Bennett-Levy J, Nagel T. Acceptability of mental health apps for aboriginal and Torres Strait islander Australians: a qualitative study. J Med Internet Res. 2016;18(3):e65. 10.2196/jmir.5314 PMC4825593 26969043

